# Major features of immunesenescence, including reduced thymic output, are ameliorated by high levels of physical activity in adulthood

**DOI:** 10.1111/acel.12750

**Published:** 2018-03-08

**Authors:** Niharika Arora Duggal, Ross D. Pollock, Norman R. Lazarus, Stephen Harridge, Janet M. Lord

**Affiliations:** ^1^ MRC‐Arthritis Research UK Centre for Musculoskeletal Ageing Research Institute of Inflammation and Ageing University of Birmingham Birmingham UK; ^2^ NIHR Biomedical Research Centre in Inflammation University Hospital Birmingham Birmingham UK; ^3^ Centre of Human and Aerospace Physiological Sciences King's College London London UK

**Keywords:** immunesenescence, inflammation, interleukin‐7, physical activity, thymic output

## Abstract

It is widely accepted that aging is accompanied by remodelling of the immune system including thymic atrophy and increased frequency of senescent T cells, leading to immune compromise. However, physical activity, which influences immunity but declines dramatically with age, is not considered in this literature. We assessed immune profiles in 125 adults (55–79 years) who had maintained a high level of physical activity (cycling) for much of their adult lives, 75 age‐matched older adults and 55 young adults not involved in regular exercise. The frequency of naïve T cells and recent thymic emigrants (RTE) were both higher in cyclists compared with inactive elders, and RTE frequency in cyclists was no different to young adults. Compared with their less active counterparts, the cyclists had significantly higher serum levels of the thymoprotective cytokine IL‐7 and lower IL‐6, which promotes thymic atrophy. Cyclists also showed additional evidence of reduced immunesenescence, namely lower Th17 polarization and higher B regulatory cell frequency than inactive elders. Physical activity did not protect against all aspects of immunesenescence: CD28^−ve^ CD57^+ve^ senescent CD8 T‐cell frequency did not differ between cyclists and inactive elders. We conclude that many features of immunesenescence may be driven by reduced physical activity with age.

## 1 INTRODUCTION

Aging is accompanied by a decline in immune competence, termed immunesenescence, which is characterized by an increased risk of infections and chronic inflammatory diseases, poor vaccine efficacy, failure to maintain immunity to latent infections such as *Varicella Zoster* and increased autoimmunity (DiCarlo, Fuldner, Kaminski & Hodes, 2009; Montecino‐Rodriguez, Berent‐Maoz & Dorshkind, 2013). The mechanisms underlying this compromised immunity include involution of the thymus, which begins in early adulthood in humans and accelerates rapidly after 40 years of age (Mitchell, Lang & Aspinall, 2010). Thymic atrophy involves a decrease in both stromal and thymocyte cellularity with infiltration of adipocytes, loss of tissue organization with the net outcome being a reduced naïve T‐cell output (Palmer, 2013). In humans, but not mice, maintenance of naïve T‐cell numbers into adulthood occurs through homeostatic proliferation and retention in secondary lymphoid tissues (Thome et al., 2016). Other hallmarks of T‐cell immunesenescence include the following: accumulation of CD28^−ve^ CD57^+ve^ T cells with shortened telomeres and reduced proliferative capacity (Di Mitri et al., 2011), which also acquire NK cell receptors such as KLRG1 (Weng, Akbar & Goronzy, 2009) increasing the risk of autoimmune responses, skewing of T‐cell responses towards Th17 cell differentiation (Ouyang et al., 2011). Although less well evidenced, some studies also suggest altered regulatory capacity with age with older adults showing increased T_reg_ frequency (reviewed in Jagger, Shimojima, Goronzy & Weyand, 2014) but lower frequency and IL‐10 production by CD19^+ve^ CD24^hi^ CD38^hi^ suppressive B cells (Duggal, Upton, Phillips, Sapey & Lord, 2013). Another feature of human aging is an increase in circulating levels of pro‐inflammatory cytokines (IL‐1β, IL‐6, TNFα,) termed *Inflammaging,* which several population‐level studies have associated with increased risk of age‐related disease and mortality (Franceschi et al., 2007).

What confounds these human studies is that physical activity is not taken into account in either cross‐sectional or longitudinal studies of immune aging. The majority of older adults are largely sedentary and fail to meet the recommended guidelines for physical activity of 150 min of aerobic exercise per week. Regular physical activity in older adults has been associated with lower levels of pro‐inflammatory cytokines such as IL‐6, TNFα (Gleeson et al., 2011), improved neutrophil chemotaxis (Bartlett et al., 2016) and NK cell cytotoxicity (Woods et al., 1999), increased T‐cell proliferation (Woods et al., 1999) and improved vaccination responses (Kohut et al., 2005). Thus, the current literature on immunesenescence is not able to determine which aspects of age‐related immune change are driven by extrinsic factors and which may be the consequence of a constitutive aging programme.

Here, we studied several aspects of the adaptive immune system in highly physically active, nonelite older individuals (master cyclists) in which we have shown the maintenance of a range of physiological functions previously reported to decline with age (Pollock et al., 2015). We show that compared with more sedentary older adults, the cyclists show reduced evidence of a decline in thymic output, inflammaging and increased Th17 cell responses, although accumulation of senescent T cells still occurred. We reveal high serum levels of IL‐7 and IL‐15 and low IL‐6, which would together provide a thymoprotective environment (Lynch et al., 2009) and also help to maintain naïve T cells in the periphery (Wallace et al., 2006). We conclude that maintained physical activity into middle and old age protects against many aspects of immune aging which are in large part lifestyle driven.

## 2 RESULTS

Immune cell phenotype was determined in peripheral blood mononuclear cells (PBMC). The sample size in each analysis varies slightly due to cell availability varying between donors and does not reflect the removal of any outlier data.

### 2.1 The effect of long‐term physical activity on T‐cell subset distribution

**Figure 1:**
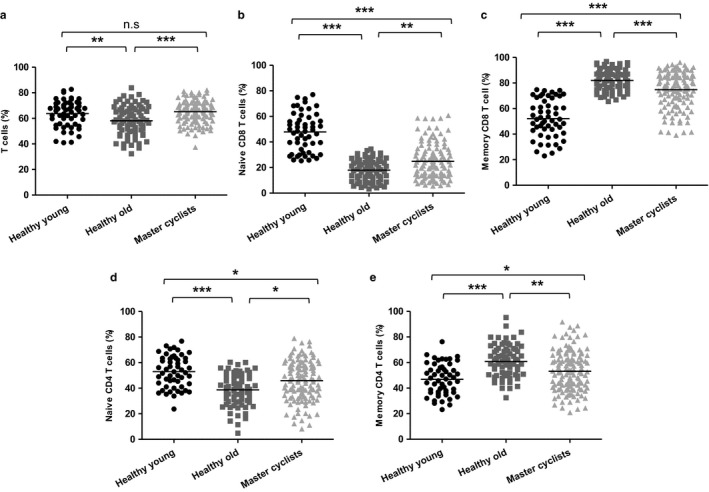
The impact of maintained physical activity on T‐cell subset distribution. Immunostaining of PBMCs shows (a) the percentage of CD3^+ve^ T cells. Further analysis of the CD3^+ve^ T cell population shows the frequency of (b) naïve CD8^+ve^ CD45RA ^+ve^ T cells and (c) memory CD8^+ve^ CD45RA ^−ve^ T cells in the CD8 T cell pool, (d) naïve CD4^+ve^ CD45RA ^+ve^ T cells and (e) memory CD4^+ve^ CD45RA ^−ve^ T cells in the CD4 pool. The solid bar represents the mean value. *p < .05, **p < .005, ***p < .001

On comparing total T‐cell frequency in the PBMC fraction between healthy young donors, healthy old sedentary donors and old master cyclists, significant differences were observed, *F*(2, 244) = 11.64, *p* < .001, β = .08. CD3^+ve^, T‐cell frequency was lower in healthy old sedentary adults compared with young donors, *p* < .005. This decline was not seen in the master cyclists as their T‐cell frequency was higher than the inactive elders, *p* = .0003 (Figure 1a) and not different from the young adults.

The frequency of naïve and memory CD8 T cells between the three groups also differed. For naïve CD8 T cells, *F*(2, 244) = 91.48, *p* = .0002, β = .42, we found a lower frequency in healthy old sedentary adults compared with young donors (*p* = .0001) indicative of the well‐documented reduction in thymic output with age (Mitchell et al., 2010; Palmer, 2013). However, naïve CD8 T cell frequency did not decline to the same extent in master cyclists being significantly higher than healthy old sedentary adults (*p* = .001), but lower than that seen in young adults, *p* = .001 (Figure 1b). The frequency of memory CD8 T cells was also different between the groups *F*(2, 244) = 90.65, *p* < .001, β = .42, due to a higher frequency in healthy old sedentary adults in comparison with young adults, *p* < .0001 and master cyclists, *p* = .0002 (Figure 1c). However, the cyclists did have a higher frequency of these cells than young subjects, *p* < .0001, suggesting that maintained physical activity did not entirely prevent the expansion of memory cells.

We similarly compared the distribution of naïve and memory CD4 T cells and memory cell subsets between the groups, and here, the differences were more marked than for CD8 cells. The frequency of naïve CD4 T cells varied across the groups, *F*(2, 244) = 16.43, *p* < .0001, β = .11, with lower values in healthy old sedentary adults in comparison with young donors, *p* = .0001. This did not occur in the cyclists who had a higher frequency of naïve cells than the sedentary older adults, *p* = .01, but a lower frequency than young subjects, *p* = .005 (Figure 1d). There was a higher frequency of memory CD4 T cells across the groups, *F*(2, 244) = 15.69, *p* < .0001, β = .11, with frequency higher in old sedentary adults compared with young donors, *p* = .0001 (Figure 1e). The cyclists had a higher frequency of CD4 memory T cells than the young subjects, *p* < .05, but the frequency was lower than for the sedentary older adults, *p* = .003.

**Figure 2:**
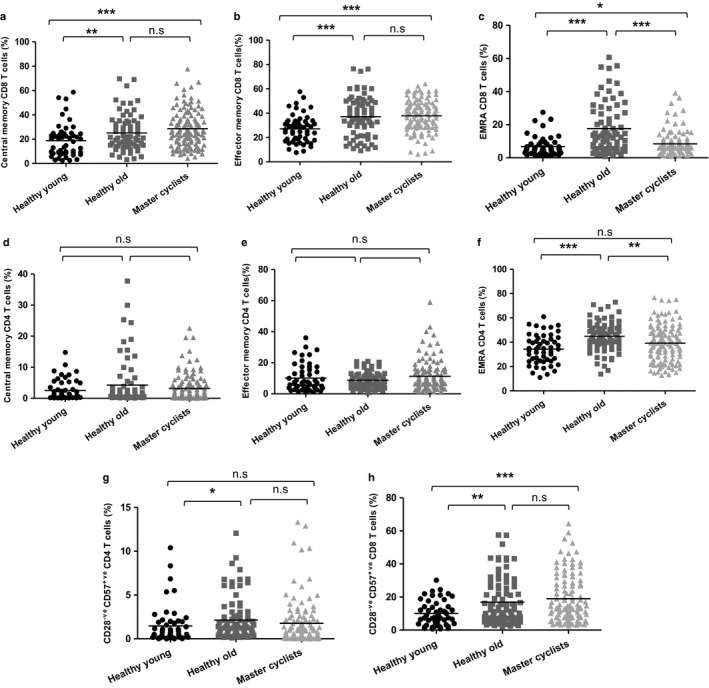
The impact of maintained physical activity on memory T‐cell subsets. Immunostaining of PBMCs shows (a) CD8 central memory (CM) characterized as CD45RA ^−ve^ CCR7 ^+ve^, (b) CD8 effector memory (EM) as CD45RA ^−ve^ CCR7^−ve^, (c) CD8 EMRA as CD45RA ^+ve^ CCR7^–ve^, (d) CD4 central memory (CM) characterized as CD45RA ^−ve^ CCR7 ^+ve^, (e) CD4 effector memory (EM) as CD45RA ^−ve^ CCR7^−ve^, (f) CD4 EMRA as CD45RA ^+ve^ CCR7^–ve^, (g) CD28^−ve^ CD57^+ve^ CD4 T cells in the CD4 pool, (h) CD28^−ve^ CD57^+ve^ CD8 T cells in the CD8 pool, in healthy young donors (*n* = 55), healthy sedentary old donors (*n* = 75) and master cyclists (*n* = 118). The solid bar represents the mean value. *p < .05, **p < .005, ***p < .001

Further analysis of the distribution of memory T‐cell phenotypes revealed that central memory CD8 T cells were at a higher frequency in old sedentary adults compared with young adults, *p* = .0001 only, with no significant differences with the master cyclists, *p* = .29. The frequency in the cyclists was also higher than the young adults, *p* < .001 (Figure 2a). For effector memory CD8 T cells, there was a significant increase in frequency in both old sedentary adults and cyclists in comparison with young donors, *p* = .0001 and *p* = .0001, respectively (Figure 2b). The differences seen between the groups in frequency of EMRA CD8 T cells were driven by a higher frequency in old sedentary adults in comparison with young donors, *p* = .0001 and master cyclists *p* = .0001, although EMRA frequency was raised in cyclists compared with young subjects, *p* < .05 (Figure 2c). There were no differences in the CD4 effector (Figure 2d) and central memory (Figure 2e) subsets between the groups although CD4 EMRA cells were raised in the sedentary old group compared with young adults, *p* < .001 and the cyclists, *p* < .005 (Figure 2f).

### 2.2 Physical activity and senescent T cells

Despite expressing telomerase to stem telomere attrition and support extensive proliferation during antigen challenge, T cells have a finite replicative potential and repeated cell division results in shortened telomeres and cell senescence (Fletcher et al., 2005). We therefore determined whether a physically active lifestyle could modify the accumulation of senescent T cells with age. CD28 and CD57 are among the cell surface markers that used to identify senescent T cells in humans (Onyema et al., 2012). On examining CD28^−ve^ CD57^+ve^ CD4 T cells, a significantly higher frequency was observed in old sedentary adults compared with young adults, *p* = .01, although cyclists did not show a raised level of these cells, *p* = .47 (Figure 2g). A much higher frequency of CD28^−ve^ CD57^+ve^ CD8 T cells was observed compared to senescent CD4 cells overall, and in both old sedentary adults and master cyclists, the frequency of senescent cells was higher compared with young adults and master cyclists, *p* = .005 and *p* = .0001, respectively (Figure 2h).

### 2.3 The effect of long‐term physical activity on B‐cell subset distribution

**Figure 3:**
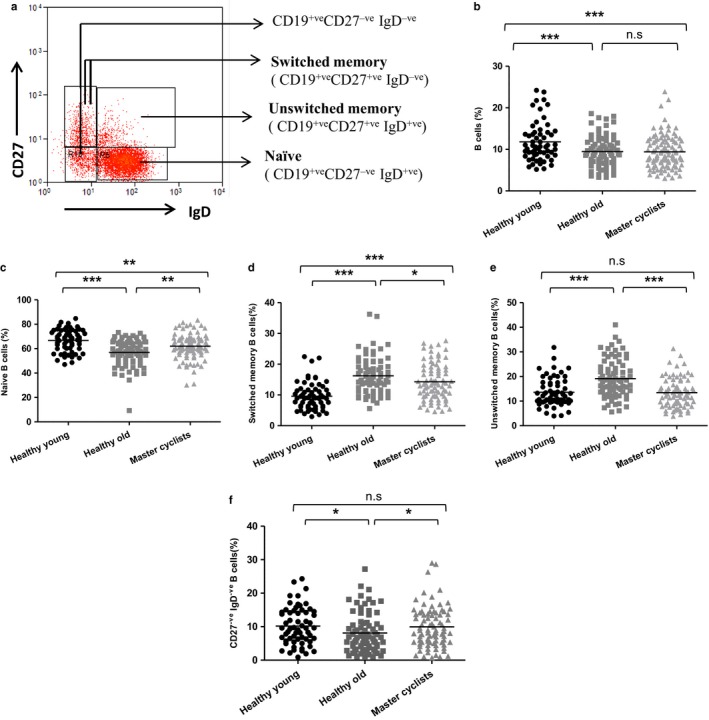
The impact of maintained physical activity on B‐cell subset distribution. (a) Representative flow cytometric plot showing the gating strategy used to identify B‐cell subsets via expression of IgD and CD27. (b) Immunostaining of PBMCs shows the frequency of CD19^+ve^ B cells. Further analysis of the B‐cell population shows the frequency of (c) naïve IgD^+ve^ CD27^−ve^ CD19^+ve^ B cells, (d) switched IgD^−ve^ CD27^+ve^ CD19^+ve^ B cells, (e) unswitched IgD^+ve^ CD27^+ve^ CD19^+ve^ B cells and (f) IgD^−ve^ CD27^−ve^ CD19^+ve^ B cells in the B‐cell pool, in healthy young donors (*n* = 55), sedentary healthy old donors (*n* = 75) and master cyclists (*n* = 108). The solid bar represents the mean value. *p < .05, **p < .005, ***p < .001

B cells can be divided into four subsets on the basis of CD27 and IgD expression (Figure 3a): naïve (CD27^−ve^ IgD^+ve^); switched memory (CD27^+ve^ IgD^−ve^) and unswitched memory (CD27^+ve^ IgD^+ve^) B cells (Shi, Agematsu, Ochs & Sugane, 2003). The CD27^−ve^ IgD^−ve^ subset is also a memory cell population (Wei et al., 2007), although much less is known of its functional significance. Comparing the peripheral B‐cell frequency between these groups, significant differences were observed, *F*(2, 238) = 12.48, *p* = .0001, β = .09, driven by a lower frequency in old sedentary adults, *p* = .0001 in comparison with young donors, with cyclists not different from the sedentary elders and showing a lower frequency than young subjects, *p* = .0001 (Figure 3b). In contrast to the naïve T‐cell data, we found a lower frequency of naive B cells in both old sedentary adults, *p* = .0001 and the master cyclists, *p* = .005 in comparison with young donors, although master cyclists had a higher frequency of naïve B cells than old sedentary adults, *p* = .003 (Figure 3c). Further, for switched memory B cells, we observed a higher frequency in old sedentary adults, *p* = .0001 and master cyclists, *p* = .0001 compared with young subjects, although frequency was lower in the cyclists than the more sedentary older adults, *p* < .05 (Figure 3d). For unswitched memory B cells, there was a higher frequency in healthy old sedentary adults in comparison with young donors, *p* = .0001 and master cyclists, *p* = .0001, with no difference between master cyclists and young adults, *p* = .89 (Figure 3e). Finally, the CD27^−ve^ IgD^−ve^ subset also differed slightly between our groups with lower frequency in the sedentary old subjects compared to both young adults, *p* = .01 and the master cyclists, *p* = .01 (Figure 3f).

In summary, the data confirm the extensive literature showing that the frequency of naive T and B cells is reduced with age, but reveal for the first time that the effect of age on naïve T‐cell populations was partially prevented in those older adults who had maintained high levels of physical activity through adult life.

### 2.4 Maintained physical activity in adulthood and thymic output

**Figure 4:**
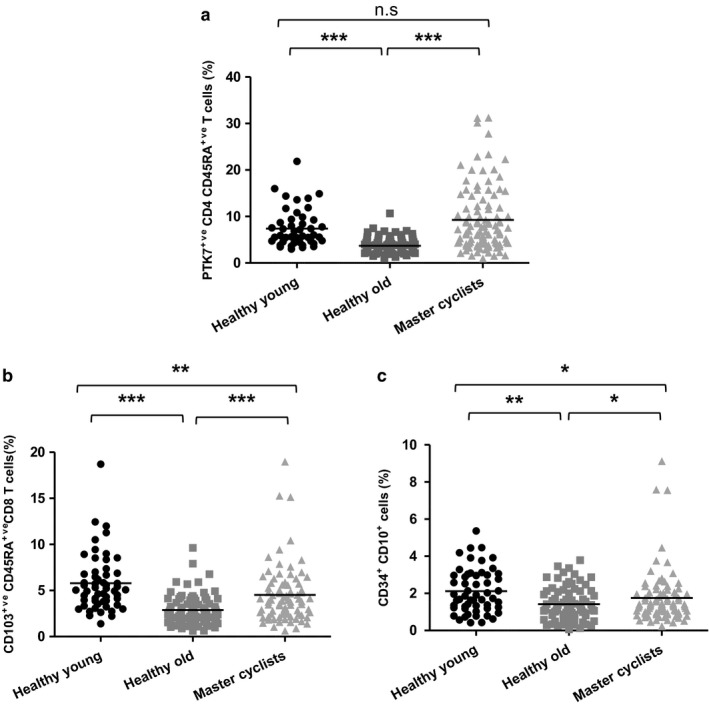
The impact of maintained physical activity on recent thymic emigrants. Immunostaining of the T‐cell population shows the frequency of (a) PTK7^+ve^ CD45RA ^+ve^ CD4 T cells in the CD4 pool, (b) CD103^+ve^ CD45RA ^+ve^ CD8 T cells in the CD8 pool, (c) CD34^+ve^ CD10^+ve^ CD19^−ve^ CD3^−ve^ CD14^−ve^ cells in the PBMC pool, in healthy young donors (*n* = 55), sedentary healthy old donors (*n* = 75) and master cyclists (*n* = 118). The solid bar represents the mean value. *p < .05, **p < .005, ***p < .001

The higher frequency of naive T cells in the master cyclists suggests that thymic output may be better preserved in these adults than their less physically active peers. PTK7 has been identified as a marker of recent thymic emigrants (RTEs), comprising CD4 T cell populations with high levels of TRECs (Haines et al., 2009). The frequency of PTK7^+ve^ CD45RA^+ve^ CD4 T cells differed between the groups, *F*(2, 242) = 24.81, *p* = .0001, β = .18, with a significantly higher frequency in master cyclists compared with old sedentary adults, *p* = .0001, but with no difference to that seen in young adults (Figure 4a).

Within the CD8 pool, CD103^+ve^ naïve cells display high TREC levels and have been identified as a population of RTEs (McFarland, Douek, Koup & Picker, 2000). The frequency of CD103^+ve^ naïve CD8 T cells differed between our groups, *F*(2, 240) = 18.55, *p* = .0001, β = .15, with a higher frequency in master cyclists in comparison with old sedentary adults, *p* = .0001, but lower than young adults, *p* = .0017 (Figure 4b).

We next wanted to determine whether the positive effects of maintained physical activity on thymic output were due to an effect on progenitor cells entering the thymus. CD3^−ve^ CD19^−ve^ CD14^−ve^ CD34^+ve^ CD10^+ve^ cells have been identified as thymic immigrants (Bender et al.*,* 1991), and their frequency differed between our groups, *F*(2, 235) = 5.25, *p* = .006, β = .04. This difference was driven by a higher frequency in master cyclists compared with old sedentary adults, *p* = .01, although the frequency in cyclists was lower than in young subjects, *p* = .01 (Figure 4c).

**Figure 5:**
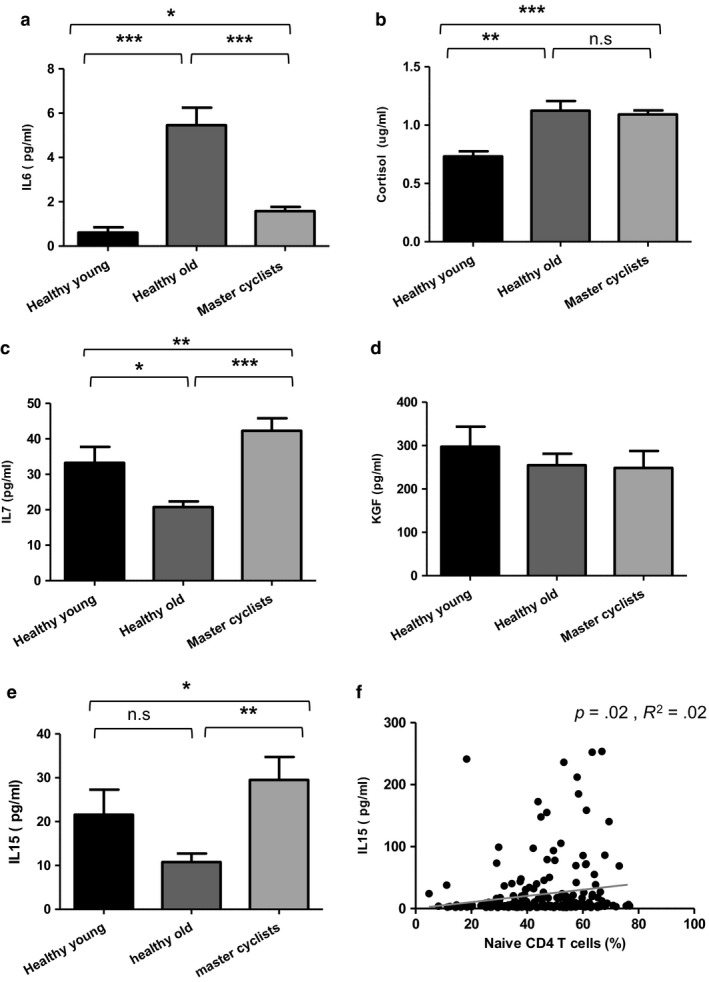
Possible mechanisms underlying maintained thymic output in cyclists. (a) Serum IL‐6 levels in master cyclists (*n* = 105), sedentary healthy old donors (*n* = 70) and young donors (*n* = 50). (b) Serum cortisol levels (ug/ml) in master cyclists (*n* = 117), sedentary healthy old donors (*n* = 75) and young donors (*n* = 52). (c) Serum IL‐7 levels in master cyclists (*n* = 119), sedentary healthy old donors (*n* = 75) and young donors (*n* = 50). (d) Serum KGF levels (pg/ml) in master cyclists (*n* = 119), sedentary healthy old donors (*n* = 75) and young donors (*n* = 50). (e) Serum IL‐15 levels in master cyclists (*n* = 119), sedentary healthy old donors (*n* = 65) and young donors (*n* = 52). In A‐E, data are mean ± *SEM*. (f) Serum IL‐15 levels were plotted against peripheral CD4 naïve T‐cell frequency (%) in master cyclists (*n* = 87). *p < .05, **p < .005, ***p < .001

These data suggest a beneficial effect of long‐term physical activity on thymic output. We thus explored factors that might be contributing towards a maintained thymic output in the cyclist cohort. On measuring serum levels of a thymosuppressive cytokine IL‐6 (Sempowski et al., 2000), significant differences were seen between the three groups, *F*(2, 224) = 12.24, *p* = .0001, β = .09, with higher IL‐6 levels in old sedentary adults compared with both young donors, *p* = .0001 and old cyclists, *p* = .0001. IL‐6 levels in the cyclists were only slightly higher than in young subjects, *p* = .04 (Figure 5a). Cortisol also affects thymic cellularity negatively (Roggero et al., 2006). Analysis of serum cortisol levels in the three groups revealed significant differences *F*(2, 241) = 12.79, *p* = .0001, β = .09, driven by higher levels in sedentary older adults in comparison with young donors, *p* = .002. However, the master cyclists also had higher cortisol levels than the young adults, *p* = .0001 (Figure 5b).

IL‐7 is a critical growth factor in early T‐cell development and is thymoprotective (Plum, DeSmedt, Leclercq, Verhasselt & Vanderkerckhove, 1996). Significant differences were observed for serum IL‐7 between the three groups, *F*(2, 244) = 9.83, *p* = .0001, β = .07, with the cyclists having significantly higher levels compared to both old sedentary adults, *p* < .0001 and young subjects, *p* < .01 (Figure 5c). Keratinocyte growth factor (KGF) is another protein important in thymus organogenesis and maintenance, and KGF administration in aged mice increases thymic size and thymocyte production (Alpdogan et al., 2006). No significant differences were seen in serum KGF levels between the groups, *F*(2, 244) = .80, *p* = .44, β = .007; (Figure 5d).

Another possible explanation of maintained naïve T‐cell populations in the cyclists is their expansion and retention in the periphery. IL‐15 contributes to the maintenance of naïve T‐cell populations by promoting survival and expansion of naïve T cells in the periphery, without leading to telomere shortening (Wallace et al., 2006). Serum IL‐15 levels in the three groups differed significantly, *F*(2, 236) = 3.30, *p* = .03, β = .03, and the master cyclists had higher levels than old sedentary adults, *p* = .001 and young adults, *p* = .01 (Figure 5e). We also found a modest positive correlation between serum IL‐15 levels and the frequency of naïve CD4 T cells *R* ^2^ = .02, *p* = .02, β = .15 (Figure 5f).

### 2.5 Th1, Th2 and Th17 cells in physically active older adults

**Figure 6:**
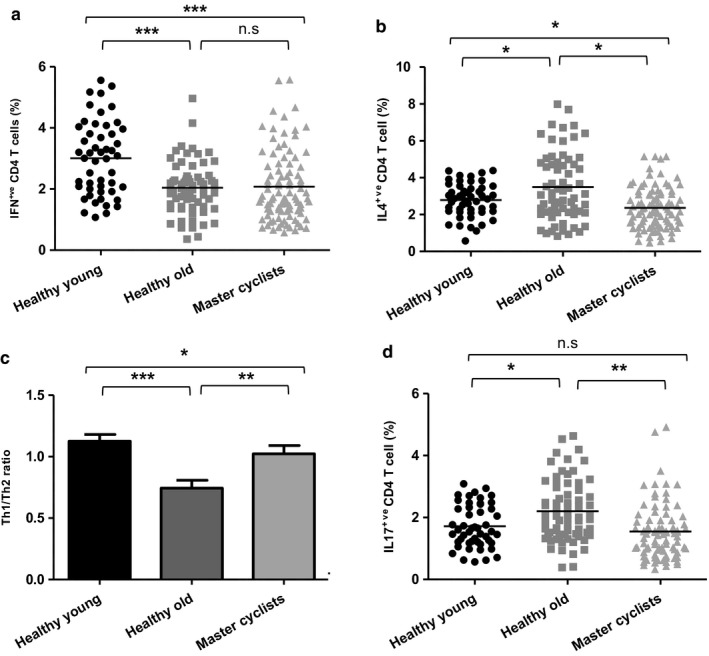
Impact of physical activity on Th1, Th2 and Th17 cell differentiation. PBMCs isolated from master cyclists, healthy young and sedentary older adults were stimulated via PMA and ionomycin for 4 hr and stained for surface expression of CD3, CD4 and intracellularly for IFNγ, IL‐4 or IL‐17. Scatter plot shows the frequency in master cyclists (*n* = 103), sedentary healthy old donors (*n* = 66) and healthy young donors (*n* = 50) of (a) IFNγ‐positive cells within the CD4 T cell subset, (b) IL‐4‐positive cells within the CD4 T cell subset, (c) shows the Th1/Th2 ratio in the same groups. (d) The frequency of IL‐17‐positive cells within the CD4 T cell subset in master cyclists (*n* = 96), healthy old donors (*n* = 70) and healthy young donors (*n* = 50). *p < .05, **p < .005, ***p < .001

Effector CD4 T cells have been classified into several subsets including Th1, Th2 and Th17 on the basis of their cytokine production. On measuring the frequency of IFNγ^+ve^ CD4 T cells (Th1) poststimulation with PMA and ionomycin, we found a lower frequency in both old sedentary adults, *p* = .0001 and master cyclists, *p* = .0001 compared to young adults (Figure 6a). Comparing the IL‐4^+ve^ CD4 T cells (Th2) poststimulation, significant differences were again seen with a higher frequency in old sedentary adults compared to young, *p* = .02, but the master cyclists had a lower frequency of Th2 cells compared to both the old sedentary adults, *p* = .006 and young adults, *p* = .007 (Figure 6b). Consequently the Th1/Th2 ratio between the groups differed, *F*(2, 216) = 7.34, *p* = .001, β = .06, with a lower ratio in old sedentary adults, *p* = .0009 and master cyclists, *p* = .008 compared to young adults (Figure 6c). The frequency of IL‐17^+ve^ CD4 T cells (Th17) poststimulation was higher in old sedentary adults compared to young subjects, *p* = .008 and cyclists, *p* = .0001, with no difference between the cyclists and young adults (Figure 6d).

### 2.6 Regulatory T and B cells

**Figure 7:**
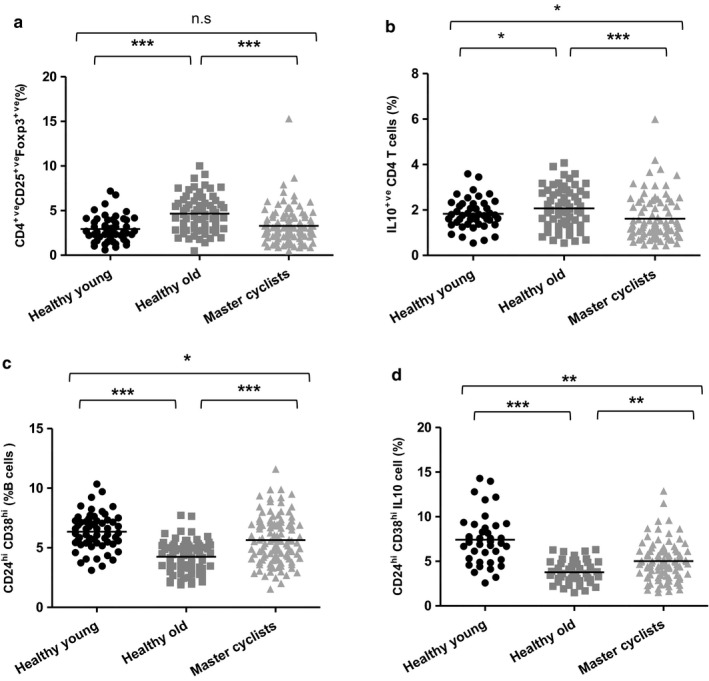
Impact of physical activity on immune regulatory cells. Immunostaining of CD4^+ve^ T cells shows the frequency of (a) CD4^+ve^ CD25^hi^ Foxp3^+ve^ T cells in healthy young donors (*n* = 52), sedentary healthy old donors (*n* = 72) and master cyclists (*n* = 120); (b) IL‐10‐positive T cells in master cyclists (*n* = 96), sedentary healthy old donors (*n* = 66) and healthy young donors (*n* = 45). Immunostaining of B cells shows the frequency (c) CD24^hi^ CD38^hi^ B cells in healthy young donors (*n* = 52), sedentary healthy old donors (*n* = 75) and master cyclists (*n* = 110), (d) IL‐10‐positive CD19^+ve^ CD24^hi^ CD38^hi^ B cells after 72‐hr stimulation in healthy young donors (*n* = 50), sedentary healthy old donors (*n* = 70) and master cyclists (*n* = 93). *p < .05, **p < .005, ***p < .001

Foxp3‐expressing CD25^+ve^CD4 T cells have been classified as regulatory T cells, T_regs_ (Hori & Sakaguchi, 2003). The frequency of these cells differed between the three groups *F*(2, 241) = 11.93, *p* < .001, β = .09, with higher frequency in old sedentary adults in comparison with young adults, *p* = .0001 and the cyclists, *p* = .0001, with no difference between the young adults and cyclists (Figure 7a). We also measured IL‐10 production by CD4 T cells poststimulation with PMA and ionomycin as a measure of regulatory potential and found differences between the three groups *F*(2, 204) = 5.25, *p* = .006, β = .05, with a higher frequency in old sedentary adults compared to young donors, *p* = .02 and old cyclists, *p* = .0001 (Figure 7b). CD19^+ve^ CD24^hi^ CD38^hi^ B cells are suggested to exert immune suppressive effects, mainly via IL‐10 secretion, which declines with age (Duggal et al., 2013). On comparing their frequency between our groups, significant differences were observed *F*(2, 234) = 27.86, *p* < .001, β = .19, driven by a lower frequency in old sedentary adults compared with young donors, *p* < .0001 and cyclists, *p* < .0001. Cyclists had slightly lower regulatory B‐cell frequency than the young subjects, *p* < .05 (Figure 7c). IL‐10 production by CD24^hi^ CD38^hi^ B cells poststimulation showed significant differences with lower IL‐10 in old sedentary adults compared with young donors, *p* = .0001 and cyclists, *p* = .001. Cyclists also had lower IL‐10 production than young adults, *p* < .005 (Figure 7d).

## 3 DISCUSSION

Remodelling of the immune system with age in humans has been assumed to be an inherent process characterized by immunodeficiency, low‐grade chronic systemic inflammation and increased risk of autoimmunity. However, the studies that have generated this assumption are confounded by not considering the impact of physical inactivity, which influences immunity and increases dramatically with age in humans. Even studies in animal models such as mice will be influenced, unless the mice are provided with the option to exercise, as laboratory‐housed animals are likely more sedentary than in the wild. This study aimed at determining to what extent the phenomenon of immunesenescence may be a consequence of our modern sedentary lifestyles and is an extrinsically driven process rather than an endogenous developmental programme.

We show here that the effect of maintained high‐level physical activity on T‐cell subset distribution is profound. One of the most striking and unexpected findings was the high frequency of CD4 and CD8 RTEs in the master cyclists, being higher than in the healthy but more sedentary older adults who had not been involved in a high level of physical activity in adulthood and equivalent to that seen in our young cohort in the case of CD4 RTEs. The thymus has been shown to be responsive to acute exercise interventions previously, with acute exercise inducing an increase in the naïve: memory T‐cell ratio in both aged mice (Woods, Ceddia, Zack, Lowder & Lu, 2003) and humans (Simpson, 2011). However, these studies did not consider RTEs and it is possible that these changes, at least in the humans, were driven by a reduction in lymphocyte margination. Here, the subjects had not exercised in the previous 24 hr, and thus, we were assessing a stable effect. The thymoprotective effect of maintained physical activity is important as reduced thymic output following thymic involution is a key element of immunesenescence affecting the ability to respond to vaccinations and novel pathogens in the environment. Moreover, the degree of reduction in thymic output has been proposed as a determinant of mortality in older adults (Ferrando‐Martínez et al., 2013).

The thymic microenvironment undergoes architectural and phenotypic changes with age including loss of stromal epithelial niches, reduced numbers of thymic epithelial cells, replacement of lymphoid tissue with adipose tissue reducing active areas of thymopoiesis (Chinn, Blackburn, Manley & Sempowski, 2012; Dixit, 2010). We have investigated the potential mechanisms by which regular exercise through adulthood exerts a positive effect on thymic output. Age‐associated reductions in factors required for thymopoiesis, such as IL‐7, have been reported in mice (Andrew & Aspinall, 2001), and IL‐7 administration in older mice (Pido‐Lopez, Imami, Andrew & Aspinall, 2002) and rhesus macaques (Aspinall et al., 2007) has been shown to enhance thymopoiesis and increase export of RTEs. Importantly, recent studies have shown that IL‐7 is produced by human skeletal muscle (Haugen et al., 2010), providing a possible explanation for raised levels in the cyclists who did not lose muscle mass with age (Pollock et al., 2015). Thymosuppressive factors, such as leukaemia inhibitory factor, oncostatin M and IL‐6, have been reported to be elevated in the aged thymus (Sempowski et al., 2000). Here, we found raised levels of serum IL‐7 and lower levels of IL‐6 in our cyclists, which would all support thymic maintenance. Furthermore, IL‐15 plays a role in regulating immune homeostasis, acting as a lymphocyte survival factor especially for naïve T cells (Wallace et al., 2006) and is also an exercise responsive hormone (Pedersen & Febbraio, 2012). Our cohort of cyclists had higher IL‐15 levels than sedentary old participants and young adults, and we observed a significant although modest positive correlation between serum IL‐15 and peripheral naïve CD4 T cells, suggesting that IL‐15 is another contributing factor towards the maintenance of the naïve cell pool in the cyclists.

Hematopoietic stem cells show an age‐associated skewed differentiation towards myeloid progenitors (Chambers, 2007) and studies in aged mice have reported fewer numbers of thymocyte progenitor cells compared to young mice (Min, Montecino‐Rodriguez & Dorshkind, 2004). Although not as well defined as in mice a population of potential thymic progenitors has been identified in humans (Bender et al., 1991), and we show here an age‐associated reduction in their frequency in the circulation, which can also be prevented with long‐term physical activity. The high level of RTEs in our cyclists is thus likely to be a result of both an increased number of thymocyte progenitor cells entering the thymus as well as an improved thymic microenvironment to promote their development and release.

Aging is also accompanied by an increase in the frequency of memory T cells and accumulation of senescent CD28^−ve^CD57^+ve^ T cells, with the effects most marked in the CD8 pool (Fletcher et al., 2005). In our study, long‐term physical activity in adulthood, with accompanying maintained thymic output, was not able to prevent the increase in the frequency of CD8 memory cells, although the increase was lower than for sedentary adults, or the accumulation of senescent CD28^−ve^, CD57^+ve^ T cells which was the same in the master cyclists and inactive elders. One cross‐sectional study has reported lower senescent T cells in physically active elders, but in that study, 52‐ to 61‐year‐olds were considered as old (Spielmann et al., 2011) which might partially explain the difference in our findings. As the accumulation of memory cells and differentiation of T cells towards the CD28^−ve^ CD57^+ve^ cells are driven largely by antigen exposure, including to latent viruses such as cytomegalovirus (Fletcher et al., 2005), it is perhaps not surprising that these immune cell changes were not counteracted by the active lifestyle of the cyclists. Indeed, analysis of CMV seropositivity in our cyclists found that 51% were CMV‐positive, which is only slightly lower than the incidence reported for adults in this age range in the United Kingdom (Vyse, Hesketh & Pebody, 2009). The analysis of the memory cell subsets revealed that the large increase in CD8 EMRA cell frequency seen in the sedentary older adults was less marked in the cyclists, despite CD28^−ve^ CD57^+ve^ cells being a component of this subset. Further analysis will be required to identify other elements of the EMRA subset which are increased in the sedentary elders but not the cyclists.

Aging is also associated with an increased potential to differentiate to a Th17 phenotype (Ouyang et al., 2011), but little is known of the effects of physical activity on Th17 cell differentiation. We found a significant decline in Th17 cells in the master cyclists and as IL‐6 plays an important role in inducing Th17 cells (Weaver, Hatton, Mangan & Harrington, 2007), the reduced serum IL‐6 in these adults may underlie the reduced Th17 skewing. Regulatory T cells play a central role in maintaining the delicate balance between pathogenic and protective immune responses. IL‐10 secretion is one mechanism used by T_regs_ to mediate immune suppression. An age‐associated increase in numbers of T_regs_ has been reported previously (Gregg et al., 2005) and was observed here in the sedentary elders, but was ameliorated in the cyclists. Wilson et al. have reported an increase in circulating T_regs_ after an acute bout of intense exercise in athletes (Wilson, Zaldivar, Schwindt, Wang‐Rodriguez & Cooper, 2009); however, this could have been mediated by reduced margination in response to adrenaline. It has been proposed that the increased number of T_regs_ with age will favour development of Th17 responses because regulatory T cells consume IL‐2 that inhibits Th17 development (O'Garra & Vieira, 2004). In our master cyclists, we saw lower peripheral T_regs_ that might in turn contribute towards the reduced Th17 polarization. The accumulation of senescent T cells, Th17 polarization, elevated levels of pro‐inflammatory cytokines and impairments in B_regs_ have all been identified as factors that might be contributing towards the age‐associated increase in risk of inflammatory autoimmune disease (Duggal et al., 2013; Goronzy & Weyand, 2012), we would thus predict reduced risk of such conditions with habitual physical activity.

The study has some limitations. The immune phenotyping data are for cell frequency only, and as lymphocyte numbers fall with age, it is possible that some of the differences between the young subjects and cyclists may be less marked for cell numbers. However, total T‐cell frequency did not decline in the cyclists suggesting this shift away from lymphoid cell generation with age is also ameliorated by physical activity. If lymphocyte numbers overall did decline in both older subject groups, this would not affect the differences between the cyclists and their inactive peers and these differences could be even more marked. Also, we do not have detailed historical physical activity data for the inactive older cohort to complement the cyclists data (Pollock et al., 2015), but we do know that they have not been involved in a high level of sports such as cycling through their adult lives and thus are a valid comparator group.

In conclusion, aging is a complex process involving the interaction of a number of factors, including genetics, environment and lifestyle. Our findings highlight that physical inactivity with age may be a profound driver of several aspects of immunesenescence, most notably reduced thymic output, changes to regulatory cell frequency and function and T‐cell polarization. Our future studies in this cohort will aim to test immune function, notably the response to vaccination, as a clinical proof of the beneficial impact of physical activity on adaptive immune function in old age.

## 4 EXPERIMENTAL PROCEDURES

### 4.1 Participant characteristics and study design

A total of 125 amateur nonelite cyclists aged 55–79 years (84 males, 41 females) were recruited. The inclusion criteria for males were the ability to cycle 100 km in under 6.5 hr while females had to cycle 60 km in under 5.5 hr, which subjects were required to have undertaken at least twice in the 3 weeks prior to testing. This cohort has been described in depth previously and had maintained their cycling activity for much of their adult lives (Pollock et al., 2015). For comparison, we also recruited adults who did not partake in regular physical activity: 75 clinically healthy older individuals aged 57–80 years (43 males, 31 females) and 55 healthy young individuals aged 20–36 years (30 males, 25 females) who were not involved in regular intense exercise. A fasting venous blood sample was taken from each subject between the hours of 08:00–10:00 after obtaining written informed consent. At the time of blood sampling, none of the subjects had an acute infection, or were taking any medication known to alter immune function, and none had any chronic illness including the older subjects. All subjects were instructed to refrain from vigorous exercise 24 hr prior to blood sampling. Serum was collected and frozen at −80°C for later hormone and cytokine analysis. Blood samples collected in vacutainers with anticoagulants were immediately processed to isolate mononuclear cells. The study was approved by the Wandsworth Research Ethics Committee (reference number 12/LO/0457) and the Black Country Research Ethics Committee (10/H1202/77).

### 4.2 Isolation of peripheral blood mononuclear cells

Peripheral blood mononuclear cells (PBMCs) were isolated by density centrifugation using Ficoll‐Paque™ PLUS (GE Healthcare, Sweden) as described previously (Duggal, Upton, Phillips, Hampson & Lord, 2014). Isolated PBMCs were frozen down by resuspending cells in freezing medium consisting of 10% DMSO (Sigma‐Aldrich, UK) in heat‐inactivated foetal calf serum (Biosera, UK), and the frozen cells were then stored at −80°C for later phenotypic and functional analysis.

### 4.3 Immunostaining to identify T‐ and B‐cell subsets

Frozen PBMCs were thawed at 37°C and washed in RPMI 1640 (Sigma‐Aldrich, UK). Postwashing, pelleted cells were resuspended in PBS (1 × 10^6^ cells/ml) and stained with combinations of antibodies: anti‐human CD3‐PEcy7 (eBiosciences, clone UCHT1); with either anti‐human CD4 Violet (eBiosciences, clone RPA‐T4) or anti‐human CD8 PE (Immunotools, clone UCHT4) was used to identify the CD4 and CD8 T cells. T cells can be classified into four distinct subsets on the basis of expression of cell surface markers, CCR7 and CD45RA; naïve (CD45RA^+ve^ CCR7^+ve^), central memory (CD45RA^−ve^ CCR7 ^+ve^), effector memory (CD45RA^−ve^ CCR7^−ve^) and effector memory RA (CD45RA^+ve^ CCR7^–ve^) using anti‐human CCR7 FITC (R and D systems, clone 150503), anti‐human CD45RA APC (Biolegend, clone HI‐100). Further phenotypes such as highly differentiated senescent T cells and recent thymic emigrants, as described in the results section or figure legends, were detected using the following antibodies: anti‐human CD28 APC (BD Biosciences, clone CD28.2), anti‐human CD57 FITC (eBiosciences, clone HCD57), anti‐human KLRG1 FITC (Biolegend, clone 2F1/KLRG1), anti‐human HLADR FITC (eBiosciences, clone H1.2F3), anti‐human CD69 FITC (eBiosciences, clone FN50), anti‐human CD25 (eBiosciences, clone BC96), anti‐human PTK7 (Miltenyi Biotech, clone׃188B), anti‐human CD103 (Miltenyi Biotech, clone Ber‐ACT8), anti‐human CD31 FITC (Biolegend, clone WM59), anti‐human CD34 FITC (eBiosciences, clone 4H11) and anti‐human CD10 APC (eBiosciences, clone CB‐CALLA) at 4°C for 20 min. For identification of B‐cell subsets, PBMCs were stained with combinations of antibodies including CD19‐PE (eBiosciences, clone׃ HIB19), CD27‐APC (eBiosciences, clone׃O323), IgD‐FITC (eBiosciences; clone׃ IA6‐2) CD24‐FITC (eBiosciences, clone׃ eBioSN3) and CD38‐PEcy7 (eBiosciences, clone HIT2). Following incubation, cells were washed and resuspended in PBS for flow cytometric analysis using a Cyan™ ADP flow cytometer (Dako). The gating strategy used to identify T‐cell subsets (Duggal et al., 2014) and Bregs (Duggal et al., 2013) has been reported previously. Data analysis was carried out using summit software. Spectral overlap when using more than one colour was corrected via compensation. Appropriate isotype controls were used for setting gates.

### 4.4 Serum cytokine and hormone‐level assays

A Bio‐plex cytokine assay for IL‐6 (Bio‐Rad Laboratories, Munich, Germany) was used for cytokine analysis using Luminex technology and performed according to manufacturer's instructions. Data acquisition and analysis were carried out using bio‐plex manager software version 6.0. Serum IL‐7, KGF and IL‐15 levels were measured by Duo Set ELISA (R and D Systems, UK) according to manufacturer's instructions. Serum cortisol levels were measured by ELISA using a commercial kit (IBL International, Germany) and according to manufacturer's instructions.

### 4.5 Stimulation of PBMCs to induce cytokine production by CD4 T cells

PBMCs were stimulated with PMA and ionomycin (both from Sigma‐Aldrich) in the presence of brefeldin A (Sigma‐Aldrich) at 37°C for 4 hr to determine the frequency of Th1 IFNγ^+ve^, Th2 IL‐4^+ve^ and Th17 IL‐17^+ve^ CD4 T cells as described previously (Duggal et al., 2014).

### 4.6 CD3 stimulation of PBMCs to induce IL‐10 production by CD19^+ve^CD24^hi^CD38^hi^ B cells

PBMCs were cultured in anti‐CD3‐coated wells for 72 hr and IL‐10 expression measured by immunostaining of CD19^+ve^CD24^hi^CD38^hi^ B cells as described previously (Duggal et al., 2013).

### 4.7 Statistical analysis

Statistical analysis was performed using the ibm spss statistics 20 software (IBM software, UK). Univariate ANOVA with least significant difference post hoc tests was used to assess differences between the three groups. A Bonferroni correction was performed to adjust for multiple comparisons. For normally distributed data, a Student's t test analysis was performed to assess differences between two conditions. Statistical significance was determined at *p* < .05.

## CONFLICT OF INTEREST

The authors have no conflict of interest to declare.

## AUTHOR CONTRIBUTIONS

NAD and RDP carried out the experimental work; NAD performed the data analysis and prepared the manuscript; and NL, SH and JML conceived the study and contributed to writing the manuscript.
